# ﻿A taxonomic synopsis and identification key to the genera of the *Myaptex* group (Diptera, Asilidae, Asilinae), with description of a remarkable new genus and three new species from the South American Chaco

**DOI:** 10.3897/zookeys.1232.142494

**Published:** 2025-03-20

**Authors:** Matheus M. M. Soares, Alexssandro Camargo, Carlos J. E. Lamas

**Affiliations:** 1 Museu de Zoologia da Universidade de São Paulo, Laboratório de Diptera, Av. Nazaré, 481, 04263-000 - Ipiranga, São Paulo, São Paulo, Brazil Museu de Zoologia da Universidade de São Paulo São Paulo Brazil; 2 Natural History Museum Vienna, 2nd Zoological Department. Burgring 7, 1010 Vienna, Austria Natural History Museum Vienna Vienna Austria

**Keywords:** Assassin flies, Chaco biome, Linnaean shortfall, Pantanal biome, robber flies

## Abstract

*Cardiasilus***gen. nov.** is erected to include the following three new species from Brazil and Paraguay: *C.aysu***sp. nov.** and *C.dangeloi***sp. nov.** (both from Mato Grosso do Sul, Brazil) and *C.ruda***sp. nov.** (Paraguay), which are herein described and illustrated. The new genus is placed into the *Myaptex* group of the subfamily Asilinae based on the following set of characters: wing with cells *r_2+3_* and *r_4_* not separated by a recurrent vein (i.e., only two submarginal cells); costal section between tips of veins *R_5_* and *M_1_* much shorter than costal section between tips of *R_4_* and *R_5_*, with *R_5_* ending bellow wing apex; scutellum tumid, with no sign of an impressed rim and with at least one pair of well-developed apical scutellar macrosetae; claws acute; and abdominal tergites with lateral marginal macrosetae. It can be differentiated from the other genera of the group by the scutum lacking anterior dorsocentral setae, male epandrium inflated (which resembles the ideogram of a heart in dorsal view), male and female sternites 2–6 with lateral macrosetae, and the male sternite 8 with a long digitiform projection at middle of posterior margin. An illustrated identification key to the genera of the *Myaptex* group, as well as to the males of *Cardiasilus***gen. nov.**, are also provided.

## ﻿Introduction

Among the Asilidae subfamilies, Asilinae Latreille, 1802 is one of the most diverse, comprising 186 valid extant genera distributed in all biogeographic regions, except Antarctica (Dikow 2020; Camargo et al. 2022, 2023). In the Neotropical region, 69 genera are recognized (Papavero 2009; Camargo et al. 2022, 2023).

To facilitate the identification of the Neotropical Asilinae genera, Artigas and Papavero (1997) proposed several generic groups delimited by morphology, including internal characters of male and female terminalia. The *Myaptex* group (Artigas and Papavero 1995) comprises nine genera (*Apulvillasilus* Camargo, Vieira & Fisher, 2022; *Atractocoma* Artigas, 1970; *Martintella* Artigas, 1996; *Myaptex* Hull, 1962; *Myaptexaria* Artigas & Papavero, 1995; *Papaverellus* Artigas & Vieira, 2014; *Rhadinosoma* Artigas, 1970; *Scarbroughia* Papavero, 2009; *Wilcoxius* Martin, 1975) and 26 species, with a somewhat disjointed distribution primarily in Central America, the Caribbean, northeastern Brazil, Chile and Argentina (Artigas and Papavero 1995; Papavero 2009; Artigas and Vieira 2014; Vieira et al. 2014; Camargo et al. 2022).

Several areas in the Neotropical region remain inadequately surveyed, particularly for Diptera, which exacerbates the Linnaean and Wallacean shortfalls. To address this gap – especially in certain regions of the Midwest and northern Brazil – the SISBIOTA-Diptera Brazilian Network research program was developed. This program aimed to document the diversity of flies in endangered and understudied areas and biomes in Brazil (Lamas et al. 2023). Most of the material examined in this study was collected during the SISBIOTA-Diptera survey, highlighting the project’s significant contribution to reducing the Linnaean and Wallacean shortfalls. It particularly aids in the revision of genera, the description of new taxa, and the recording of new distribution data.

The purpose of this paper is to describe and illustrate a new genus of Asilinae (*Myaptex* group), with three new species from Brazil and Paraguay. Additionally, we provided a taxonomic synopsis of fauna, together with remarks for the genera, an illustrated identification key for the *Myaptex* group genera and a key to species for the males of the new established genus *Cardiasilus* gen. nov.

## ﻿Materials and methods

The studied specimens are housed at Museu de Zoologia da Universidade de São Paulo (**MZUSP**) and Natural History Museum Vienna (**NHMW**), with photographs of specimens held at the California Academy of Sciences (**CAS**). Terminology follows mainly Cumming and Wood (2017). Male terminalia were dissected with the abdomen cut at the beginning of the sixth segment. The dissected parts were macerated with potassium hydroxide (KOH at 10%) and left at room temperature for seven days. Subsequently, they were neutralized in baths of tap water for 10 min and acetic acid at 10% for 30 min. Then, the dissected parts were transferred to an excavated slide containing glycerin for visualization, analyses, and imaging of its structures under a stereomicroscope. After being examined, the terminalia pieces were placed in a microvial with glycerin and pinned under the corresponding specimen.

Photographs were obtained using the Zeiss® Discovery V20 stereomicroscope with a Zeiss AxioCam Mrc5 camera attached, connected to a desktop computer through Zeiss AxioVs40 v. 4.8.2.0 software. Image sequences were assembled in Helicon Focus 6.7.1 software, with some further editing with Adobe Photoshop. Label data for the primary types are cited verbatim in quotation marks (each line separated by a vertical line “|” and each label by a semicolon “;”), with annotations in square brackets. Distribution map was created with Simplemappr (Shorthouse 2010), using the data present on the specimen labels.

## ﻿Results

### ﻿Identification key to the genera of the *Myaptex* group (Asilinae)

**Table:** 

1	Wings with bifurcation of *R_4_* and *R_5_* before the apex of discal cell (*d*) (Figs 2G, 6F, 9F)	**2**
–	Wings with bifurcation of *R_4_* and *R_5_* at or after the apex of discal cell (*d*) (Figs 13B, 17B)	**3**
2	Abdominal sternites with at least 3 pairs of macrosetae (Figs 2H, 6E, 9E); epandria inflated, resembling the ideogram of a heart (i.e., Figs 2B, H, 3A, C). Length, 10–13 mm (Brazil, state of Mato Grosso do Sul and Paraguay)	***Cardiasilus* gen. nov.**
–	Abdominal sternites without macrosetae (Artigas and Vieira 2014: fig. 1); epandria not inflated and flat ventrally, resembling a goat hoof (Artigas and Vieira 2014: fig. 1). Length, 15 mm (Brazil, states of Pará and Piauí)	***Papaverellus* Artigas & Vieira, 2014**
3	Body covered with abundant squamiform-fusiform setae (Fig. 1A, B; Camargo et al. 2022: figs 1, 2)	**4**
–	Body without squamiform-fusiform setae (i.e., Fig. 2A, B)	**5**
4	Frons with divergent slopes (Camargo et al. 2022: fig. 3); empodium and pulvillus absent (Camargo et al. 2022: fig. 4). Length, 6–9 mm (Argentina)	***Apulvillasilus* Camargo, Vieira & Fisher, 2022**
–	Frons with parallel slopes (Fig. 1D); empodium and pulvillus present (Fig. 1A). Length, 12 mm (Chile)	***Atractocoma* Artigas, 1970**
5	Sternites with macrosetae (Figs 13E, 15E, 18E)	**6**
–	Sternites without macrosetae (Fig. 16E)	**8**
6	Postpedicel lanceolate, approximately as long as scape and pedicel combined (Fig. 18A, C); mystax restricted to lower 1/2 of face, composed by long and sparse macrosetae (Fig. 18C, D); sternites with 2 pairs of macrosetae (Fig. 18E); phallus exposed (Fig. 18F) Length, 10–11 mm (Cuba, Mexico, Dominican Republic, Guatemala, Honduras, El Salvador, Nicaragua)	***Wilcoxius* Martin, 1975**
–	Postpedicel oval, shorter than scape and pedicel combined (Figs 13C, 15C); mystax occupying almost the entire face, composed by long and dense macrosetae (Figs 13C, D, 15C, D); sternites with abundant setae (Figs 13E, 15E); phallus completely concealed (Figs 13F, 15F)	**7**
7	Scutellar disc only with scattered, long setae (Fig. 13B); normally 2–4 black apical scutellar macrosetae; male terminalia with epandria strongly inflated, their apices curved in apically (Fig. 13F). Length, 8–13 mm (Chile)	***Myaptex* Hull, 1962**
–	Scutellar disc with two tufts of abundant, proclinate, long setae (Fig. 15B, C); from 2 to several apical scutellar macrosetae (sometimes mixed black and white) (Fig. 15C); male terminalia with epandria not inflated, their apices blunt and not curved in at apex (Fig. 15F). Length, 17–19 mm (Chile)	***Myaptexaria* Artigas & Papavero, 1995**
8	Frons with converging slopes in anterior view (Fig. 12A); mystax restricted to middle of face, resembling a mohawk (Fig. 12A, B); base of vein *R_4_* usually nearly straight (Vieira et al. 2014: figs 19, 31) (Costa Rica, Mexico, and Trinidad and Tobago)	***Martintella* Artigas, 1996**
–	Frons with parallel slopes in anterior view (Figs 16C, 17E); mystax not restricted to the middle of face and not resembling a mohawk (Figs 16C, 17D); base of vein *R_4_* angulated (Fig. 17B)	**9**
9	Mystax composed of few sparse macrosetae (Fig. 16C, D); at least one pair of well-developed anterior dorsocentral macrosetae (Fig. 16A, D); vein *R_4_* ending at wing apex (Fig. 16B) (Chile)	***Rhadinosoma* Artigas, 1970**
–	Mystax composed of abundant and dense macrosetae (Fig. 17C, D); well-developed anterior dorsocentral macrosetae absent (Fig. 17A); vein *R_4_* ending before wing apex (Fig. 17B) (Mexico)	***Scarbroughia* Papavero, 2009**

### ﻿Taxonomic synopsis

#### ﻿ Apulvillasilus

Taxon classificationAnimaliaDipteraAsilidae

Camargo, Vieira & Fisher

638FC03E-BE68-5F93-B410-DDBE15DC16CD

Apulvillasilus Camargo, Vieira & Fisher, 2022. Type species: Apulvillasilusboharti Camargo, Vieira & Fisher, 2022 (original designation). Type locality: Argentina, Catamarca Province, Belén.

##### Remarks.

The monotypic genus *Apulvillasilus* was recently described and illustrated from Argentina. The genus shares with *Atractocoma* Artigas the body covered with abundant squamiform-fusiform setae but can be easily differentiated by the frons with divergent slopes and the legs lacking empodium and pulvillus (a unique condition among the *Myaptex* group).

##### Distribution.

Argentina.

#### ﻿ Atractocoma

Taxon classificationAnimaliaDipteraAsilidae

Artigas

C25A0E6A-72B8-5415-A4C4-CE78645FDD09

Fig. 1

Atractocoma Artigas, 1970. Type species: Atractocomanivosa Artigas, 1970 (original designation). Type locality: Chile, Aysén.

##### Remarks.

**Figure 1.**
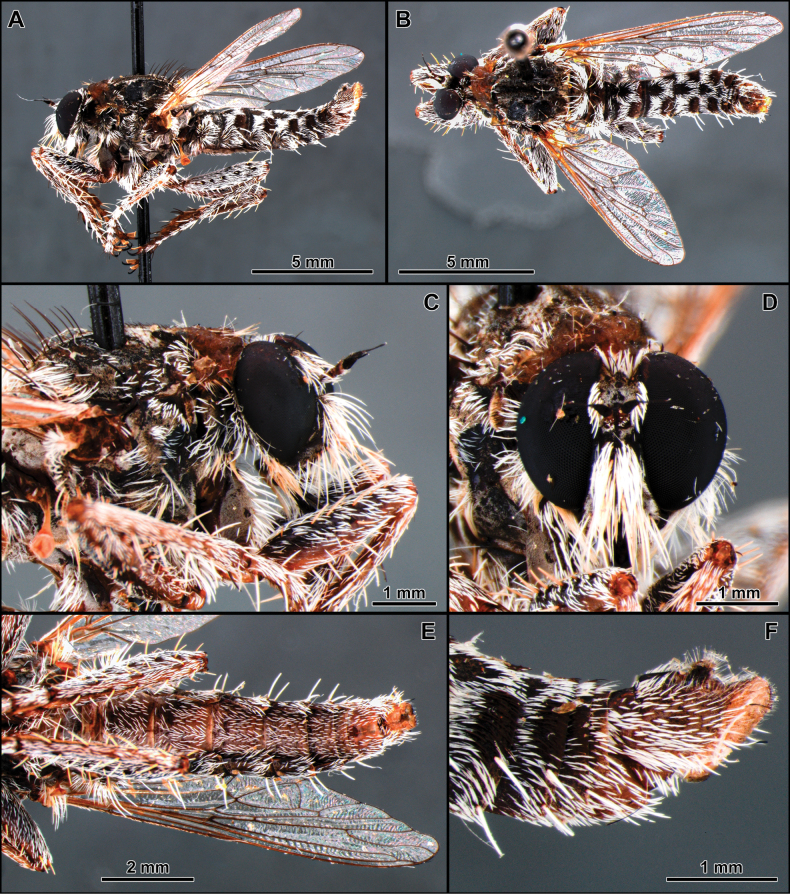
*Atractocomanivosa* Artigas, 1970 (identified male from Chile) **A, B** habitus lateral and dorsal, respectively **C** head and thorax, lateral view **D** head, anterior view **E** abdomen, ventral view **F** terminalia, lateral view.

The monotypic genus *Atractocoma* is known only from Chile. It shares with *Apulvillasilus* the body covered with abundant squamiform-fusiform setae (as mentioned above) (Fig. 1A, B) but can be easily differentiated by the dense mystax, occupying almost the entirely face (Fig. 1C, D); scutum lacking distinct rows of acrostichal setae and anterior dorsocentral setae absent (Fig. 1A, B); scutellum with three pairs of apical macrosetae and dorsally covered with long and abundant white setae; tergites 2–6 in dorsal view with V-shaped pattern formed by squamiform-fusiform setae (Fig. 1B) and sternites lacking macrosetae (Fig. 1E).

##### Examined material.

Chile, Chico, Lag. Buenos Aires, Aysen, CHILE, 24–31.xii.1960, Pena (1 ♂, 1 ♀, MZUSP).

##### Distribution.

Chile.

#### ﻿ Cardiasilus gen. nov.

Taxon classificationAnimaliaDipteraAsilidae

3FC0DF61-0E4E-5559-89AC-AE111A6F0E03

https://zoobank.org/CC0B7CD3-B621-45A9-8B0A-1852110EE91C

Figs 2 , 3 , 4 , 5 , 6 , 7 , 8 , 9 , 10 , 11 , 19

##### Type species.

*Cardiasilusaysu* sp. nov. by present designation.

##### Etymology.

From the Greek feminine word *kardia* = heart + *asilus* = common epithet of robber flies. The name refers to the distinct inflated epandria, somewhat heart ideogram-shaped in dorsal view. The gender is masculine.

##### Diagnosis.

**Figure 2.**
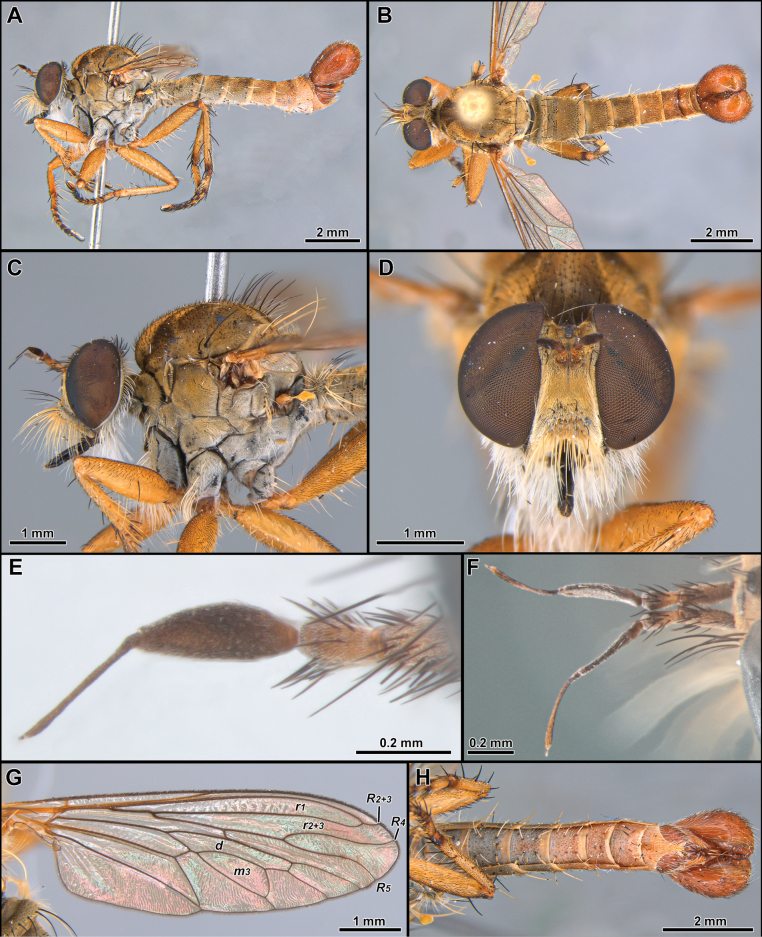
*Cardiasilusaysu* sp. nov. (male holotype) **A, B** habitus lateral and dorsal, respectively **C** head and thorax, lateral view **D** head, anterior view **E, F** antenna, lateral and dorsal views, respectively **G** wing **H** abdomen, ventral view. Abbreviations: *d* = discal cell; *m3* = third medial cell; *r1* = first radial cell; *r2+3* = second + third radial cell; *R2+3* = second branch of radius; *R4* = upper branch of third branch of radius; *R5* = lower branch of third branch of radius.

**Figure 3.**
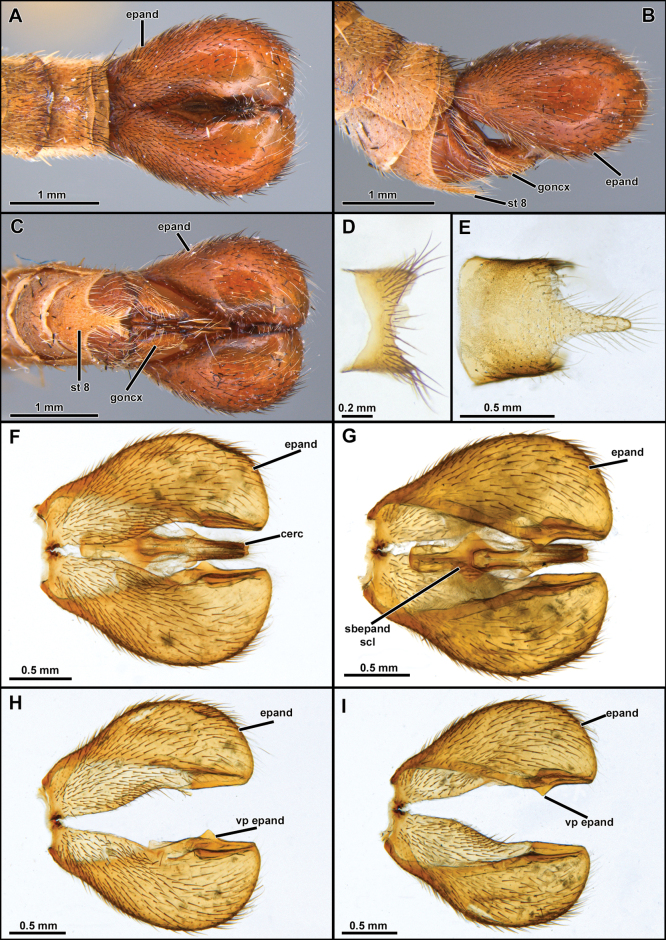
*Cardiasilusaysu* sp. nov. (**A–C** male holotype **D–I** male paratype) **A–C** terminalia in dorsal, lateral, and ventral views, respectively **D** tergite 8 **E** sternite 8 **F, G** dissected terminalia in dorsal and ventral views, respectively **H, I** dissected terminalia without inner appendages in dorsal and ventral views, respectively. Abbreviations: cerc = cercus; epand = epandrium; goncx = gonocoxite; sbepand scl = subepandrial sclerite; st 8 = sternite 8; vp epand = ventral process of epandrium.

***Head*.** Antenna ~ 3/4 as long as eye height (Fig. 2A). Scape and pedicel subequally long (Fig. 2E). Postpedicel lanceolate, laterally compressed and slightly tapering towards apex, about as long as scape and pedicel combined (Fig. 2E). Stylus slightly longer than postpedicel, composed of two elements (Fig. 2E). Face wide, slightly narrowing at antennal level and slightly gibbous at lower 1/3, mystax restricted to gibbosity (Fig. 2D). Frons with parallel slopes, slightly concave at antennal level, twice wider than higher (Fig. 2D). Palpus one-segmented, short, ~ 1/5 length of proboscis. Proboscis ~ 3.5/5 as long as eye height (Fig. 2C). ***Thorax***. Acrostichal setae indistinct (Fig. 2C). Anterior dorsocentral macrosetae absent, scutum with three to four pairs of posterior dorsocentral macrosetae (Fig. 2C). Scutellum tumid with pair of marginal macrosetae (Fig. 2B). Anatergite bare. Postmetacoxal area membranous. ***Legs.*** Femora swollen. ***Wing.*** Distinctly shorter than abdomen, with bifurcation of veins *R_4_* and *R_5_* placed before apex of discal cell by approx. length of *r-m* cross vein (Fig. 2G). Distance between apex of veins *R_4_* and *R_5_* ~ 1.5–2× longer than distance between apex of veins *R_5_* and *M_1_* (Fig. 2G). Cells *m_3_* and *cua* closed and petiolate before wing margin (Fig. 2G). ***Abdomen***. Abdominal sternites 2–6 with two to three pairs of pale yellow macrosetae mid-laterally (Figs 2H, 6E, 9E). Sternite 8 with mid-posterior digitiform projection almost as long as sternite 8 length (Figs 3E, 7E, 10E). ***Terminalia***. Epandria inflated laterally and posteriorly, resembling the ideogram of heart in dorsal view. Phallus long and thin, longer than length of hypandrium plus gonocoxite, divided into two prongs along its entire length (i.e., Fig. 3A–C, F–I).

##### Remarks.

*Cardiasilus* gen. nov. is similar to *Myaptex* Hull by the inflated male epandria (Fig. 14A–D), but can be easily distinguished by the following set of characters: postpedicel lanceolate, approx. as long as length of scape and pedicel combined (Fig. 2E, F); face slightly gibbous at lower 1/3 (Fig. 2C); scutum lacking distinct rows of acrostichal setae; anterior dorsocentral setae absent; femora mostly covered with short black setae (Fig. 2C); male sternite 8 with a long digitiform projection at posterior margin (Figs 2H, 3C, E); gonocoxite L-shaped, with rounded apex and covering the gonostylus versus postpedicel oval, shorter than scape and pedicel combined (Fig. 13C); face distinctly gibbous at lower 2/3 (Fig. 13C); scutum with two distinct rows of acrostichal setae (Fig. 13A, C); three to four pairs of anterior dorsocentral macrosetae; femora mostly covered with long vestiture of white setae (Fig. 13A); male sternite 8 without projections at posterior margin; gonocoxite squared at base, with a digitiform apicoventral projection acute apically (Fig. 14F) in *Myaptex*. It is also similar to some undescribed *Lecania* Macquart species, including *Neraxeurylabis* (Wiedemann, 1828) a species that belongs in *Lecania* (unpublished data) with inflated male epandria, but can be easily distinguished by the scutellum with one pair of apical macrosetae and the abdominal sternites with macrosetae (scutellum bare in *Lecania* or at most bearing tiny, short setulae and sternites only with sparse setae).

##### Distribution.

The new genus is known to occur only in the state of Mato Grosso do Sul (Central-West Brazil) and department of Cordillera (Central-West of the Oriental Region of Paraguay), in biomes of Pantanal and Chaco, respectively (Fig. 19).

### ﻿Key to species of *Cardiasilus* gen. nov. (males)

**Table:** 

1	Inner dorsal margin of epandrium with a short, sub-triangular, pre-apical, process followed by a short, ventral, sub-rectangular, anteriorly curved, apical process (Fig. 10A, F, G). Subepandrial sclerite goblet-shaped (Figs 10F, G, 11D) (Paraguay: Cordillera)	***Cardiasilusruda* sp. nov.**
–	Inner dorsal margin of epandrium lacking processes, only ventral margin with a short, pre-apical process (Figs 3A, F–I, 7A, F–I). Subepandrial sclerite somewhat trapezoidal or diamond-shaped (Figs 4D, 8D) (Brazil: Mato Grosso do Sul)	**2**
2	Anterior row of macrosetae on mid femur wholly white (rarely with 1 or 2 black macrosetae at apical 1/2); postalar macrosetae yellow (Fig. 2C) (sometimes 1 postalar macroseta black); epandrium weakly excavated at mid-inner dorsal margin (Fig. 3A, H); subepandrial sclerite somewhat diamond-shaped near its middle (Fig. 4D)	***Cardiasilusaysu* sp. nov.**
–	Anterior row of macrosetae on mid femur wholly black, only the basalmost macroseta white (Fig. 6A); postalar macrosetae black (Fig. 6B); epandrium strongly excavated at mid-inner dorsal margin (Fig. 7A, 7F); subepandrial sclerite somewhat trapezoidal at its middle (Fig. 8D)	***Cardiasilusdangeloi* sp. nov.**

#### ﻿ Cardiasilus aysu sp. nov.

Taxon classificationAnimaliaDipteraAsilidae

DC34FA99-F5D6-58A5-9A03-73012AB2BA86

https://zoobank.org/FD78363D-6751-4BEC-BC76-F3AD67423224

**Figure 4.**
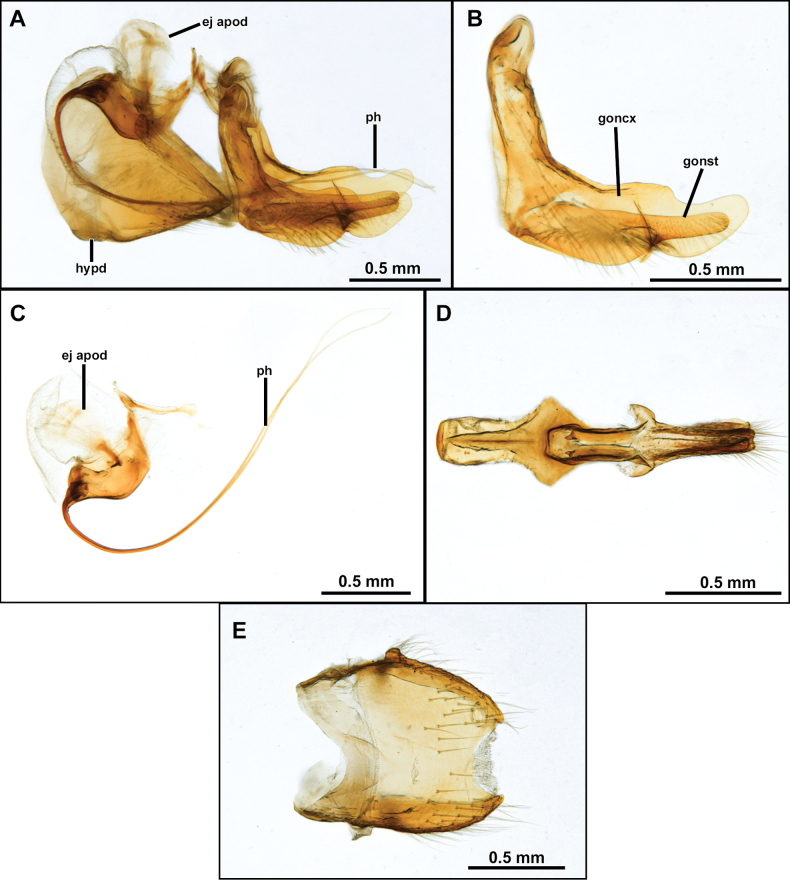
*Cardiasilusaysu* sp. nov. (male paratype) **A** inner appendages **B** gonocoxite and gonostylus **C** phallus and ejaculatory apodeme **D** subepandrial sclerite **E** hypandrium. Abbreviations: ej apod = ejaculatory apodeme; hypd = hypandrium; goncx = gonocoxite; gonst = gonostylus; ph = phallus.

**Figure 5.**
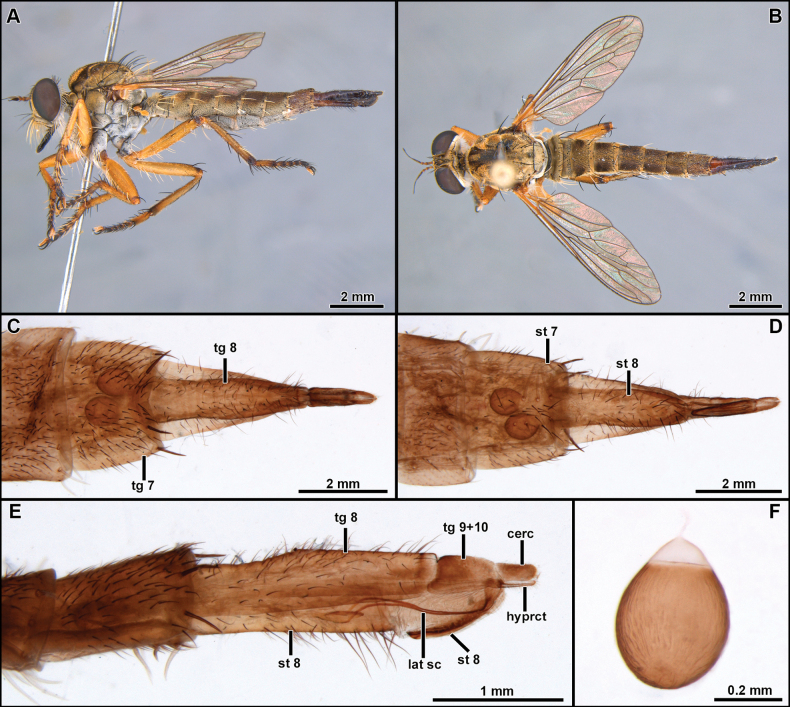
*Cardiasilusaysu* sp. nov. (identified female) **A, B** habitus lateral and dorsal, respectively **C–E** terminalia in dorsal, ventral, and lateral views, respectively **F** spermathecae. Abbreviations: cerc = cercus; hyprct = hypoproct; lat sc = lateral sclerite of genital fork; st = sternite; tg = tergite.

Figs 2 , 3 , 4 , 5 , 19

##### Diagnosis

**(males).** The new species can be easily distinguished from the congenerics by the yellow postalar macrosetae (Fig. 2B, C), inner dorsal margin of epandrium weakly excavated, and lacking processes (Fig. 3A, F, H) and subepandrial sclerite somewhat diamond-shaped near its middle (Fig. 4D).

##### Description.

**Male holotype** (Fig. 2A). Body Length 12.3 mm; wing length 6.8 mm. ***Head*** (Fig. 2A–F). Scape and pedicel orange-yellow, slightly darker at apex; postpedicel dark brown, lanceolate; stylus dark brown, bare, first element very short, second element long and abruptly narrowed at apex. Face golden pruinose, except gibbosity with mixed silvery and golden pruinosity; mystax with few upper slender black macrosetae and strong golden macrosetae below, extending along oral margin. Frons, golden pruinose, with long black orbital macrosetae. Ocellar tubercle dark brown, with one to two pairs of proclinate long and slender ocellar macrosetae; vertex golden pruinose, almost bare of major setae, only with few slender black setae posteriorly; upper-most four to five postocular macrosetae black, remaining macrosetae yellowish-white. Postcranium golden pruinose, with long, slender, and dense white lower occipital setae. Palpus dark brown, with few short white setae. Proboscis black, ventral surface with long white setae, apex with short white setae. ***Thorax*** (Fig. 2A–C). Antepronotum with few strong golden macrosetae, covered with golden pruinosity and sparse short and white setae. Scutum wholly covered with short vestiture of black setae and golden pruinosity, except notopleural, supra-alar, and acrostichal area close to scutellum with short white setae, in anterior view with U-shaped dark brown median stripe along acrostichal area, followed by L-shaped dark brown paramedian stripe along dorsocentral and intra-alar areas, with both stripes extending slightly beyond transverse suture. Three to four pairs of black, postsutural dorsocentral macrosetae, two black notopleural macrosetae, one black supra-alar macroseta, two yellow postalar macrosetae (anterior one shorter). Scutellum with one pair of apical pale yellow macrosetae, dorsal surface covered with short white setae. Pleuron mostly silvery pruinose, except anepisternum and katepisternum with golden pruinosity anteriorly, anepisternum, katepisternum, anepimeron and meron with few long white setae posteriorly, katatergite with vertical row of yellow macrosetae. ***Legs*** (Fig. 2A). Mostly yellow, except all coxae with dense silvery pruinosity, all femora dorsally with short black setae, apex of mid and hind femora dorsally, apex of all tibiae, apex of all tarsomeres one to four and all tarsomeres five dark brown. Legs with white or golden macrosetae, except when as noted. **Leg I.** Coxa with long and dense white macrosetae anteriorly. Femur with one posterior black macroseta at middle, ventral surface with long setae at basal 1/2. Tibia with one anterodorsal black macroseta at basal 1/4, three dorsal black macrosetae, one near middle and two at apical 1/2, one antero, one dorsal, one posterodorsal, and one ventral black macrosetae at apex, two anterior long macrosetae at apex (~ 2× longer than remaining apical macrosetae), posterior row of four long macrosetae from basal 1/4 to apex, three ventral long black setae near middle. Basitarsus with one antero and one posteroventral short black macrosetae at apex, one posteroventral long macroseta near base, tarsomeres one to three with crown of long macrosetae at apex: one antero and one posteroventral, one antero and one posterodorsal. **Leg II.** Coxa with long and slender macrosetae at apical 1/2, some of them reaching lateral surface. Ventral surface of femur with short vestiture of white setae and five to six long ventral macrosetae at basal 1/2, one anterodorsal apical short black macroseta, one posterodorsal preapical short macroseta, anterior row of four macrosetae from base to apex, anteroventral row of short intermixed black and white macrosetae. Tibia with two to three short dorsal black macrosetae at apical 1/2, one ventral long black macroseta at middle, ventral row of short and slender black setae from basal 2/4 to apex, one posterior long and slender black seta at basal 1/3, two ventral strong macrosetae, one black at middle and one white preapical, one posterior strong macroseta at apical 1/3, crown of long intermixed black and white macrosetae at apex: one antero and one posteroventral, one antero and one posterodorsal, one anterior and one posterior. Tarsus with chaetotaxy similar to fore tarsus. **Leg III.** Coxa laterally with two posterior macrosetae. Femur with two to three anterior macrosetae, anteroventral row of short macrosetae, posteroventral row of long and slender setae at basal 1/2, few long and slender posterodorsal setae near base, one anterodorsal black preapical macroseta, one dorsal preapical macroseta. Tibia with one antero and one posterodorsal short black macrosetae near base, two long anterodorsal black macrosetae: one near middle and one at apical 1/2, two long anteroventral black macrosetae at apical 1/2, crown of black macrosetae at apex: one antero and one posteroventral, one ventral, one anterior and one dorsal. Tarsus with chaetotaxy similar to fore and mid tarsus. ***Wing*** (Fig. 2G). Hyaline, veins brown, orangish at base and Sc. Membrane with sparse dark brown microtrichia at apex of cells *r_1_*, *r_2+3_*, *r_4_* and bordering the veins *R_4_* and *R_5_*. Halter: yellow. ***Abdomen*** (Fig. 2A, B, H). Mostly brown, becoming orange from segments five to eight, densely covered with golden pruinosity, except lateral margins of tergites one to four, silvery pruinose. Posterior margin of tergite one with six to seven long black macrosetae, lateral margin with seven to eight white lateral marginal macrosetae, lateral margins of tergites two to six with two strong white lateral marginal macrosetae, tergites mostly clothed by short vestiture of black setae, becoming white laterally. Sternites one to four densely silvery pruinose, sternites five to eight mostly orange, with weak silvery pruinosity, sternites two to six with two to three pairs of pale yellow macrosetae mid-laterally and clothed with short, sparse, white setae. ***Terminalia*** (Figs 3, 4). Orange-brown. Tergite eight somewhat saddle-shaped, narrowing at middle of anterior and posterior margins, posterior 1/2 with two to three rows of short setae, longer at posterior corners (Fig. 3D). Sternite eight with short and slender white setae at posterior 1/3, with long digitiform projection at middle of posterior margin, ~ 2/3 as long as sternite eight length, with conspicuous yellow setae (Fig. 3E). Epandrium inflated laterally and posteriorly, resembling the ideogram of a heart in dorsal view, lacking inner and apical projections dorsally, inner ventral margin with a short preapical dentiform process, mostly with short vestiture of brownish setae, except apex with slightly longer yellowish setae (Fig. 3A–C, F–I). Cercus short, digitiform, laterally compressed, covered with short setae (Fig. 3A). Subepandrial sclerite long and narrowing towards apex, with a median subtriangular lateral process (somewhat diamond-shaped near its middle), almost at the same level, internally, with a pair of short, tooth-like processes directed anteriorly, and with a short dentiform preapical process, apex dorsoventrally flattened and covered with short setae (Fig. 4D). Hypandrium subrectangular, with a short concavity at posterior margin, covered with short setae at posterior 1/2 (Fig. 4E). Gonocoxite L-shaped, pointed at base and rounded at apex, with slightly preapical concavity at dorsal margin, few short setae at basal 2/3 of external surface (Fig. 4A, B). Gonostylus ~ 1/2 as long as gonocoxite, somewhat digitiform, with ventral indentation at apical 1/3, apex rounded (Fig. 4A, B). Ejaculatory apodeme fan-shaped (Fig. 4C). Phallus long and thin, longer than length of hypandrium plus gonocoxite, divided into two prongs along the entire length (Fig. 4C).

**Female** (Fig. 5). Similar to male, except as noted: Body length 13.5 mm; wing length 6.8 mm. Abdomen wholly brown, only the segment seven and the basal 1/2 of ovipositor sometimes orange, tergite seven with dark brown macrosetae posteriorly, sternite seven with white macrosetae laterally and posteriorly. ***Terminalia*** (Fig. 5C–F). Laterally compressed from middle of tergite and sternite eight, long and slender, almost two times the length of tergite seven, covered with short black setae, longer before the opening of the genital fork, apex of sternite eight curved ventrally, weakly sclerotized, bare, and strongly striated. Tergite 9+10 slightly longer than cercus, mostly bare and shiny, only covered with few short and sparse white setae. Cercus short, digitiform, covered with short and slender white setae. Hypoproct short, V-shaped. Two sclerotized and rugose spermathecae present, membranous at base.

##### Type examined material.

***Holotype***: • ♂ (MZUSP) labelled: “Brasil: MS [state of Mato Grosso do Sul]: Porto Murtinho | Trilha Fazenda Campo Florido | 21°41'52,0"S, 57°45'57,1"W | Ativa | 11.xii.2013 | Lamas & eq. cols. | SISBIOTA | CNPq/FAPESP” “HOLOTYPE | *Cardiasilusaysu* | Soares, Camargo & Lamas [red label]”. Holotype condition: Good, not dissected. ***Paratypes***: • same data as holotype (2 ♂, one dissected, MZUSP); same data, except: 21°38'15,07"S, 57°42'10,2"W | Coleta manual (rede) | 12.xii.2011 | Lamas, Nihei & eq. col. (1 ♂, MZUSP); same data, except: Fazenda Retiro Conceição – Trilha | da Espinhadeira | 21°40'59,7"S, 57°46'42,5"W | Malaise 31 | 10–25.i.2012 | Lamas, Nihei & eq. col. (2 ♂, MZUSP); same data, except: 21°41'06.2"S, 57°46'35.7"W | coleta manual (rede) | 11.xii.2011 (1 ♂, MZUSP; 2 ♂, NHMW); • Porto Murtinho (norte) | Estrada para Pirizal, km18 | 21°33'05"S, 57°45'35"W | 19–31.i.2008 | Nihei, S.; Figueiredo, R. & Almeida, J. (col.) (2 ♂, MZUSP; 2 ♂, NHMW); • same data, except: 21–30.i.2008 (2 ♂, one dissected, MZUSP); same data, except: 24.i.2008 | F.A. Esteves col. (1 ♂, MZUSP); • same data, except: Faz. São Fernando, km12 | 21°36'30"S, 57°49'02"W | 19–31.i.2008 | Nihei, S.; Figueiredo, R. & Almeida, J. | (col.) (1 ♂, MZUSP); same data, except: Arredores do Hotel dos Camalotes | 21°42'28"S, 57°35'00"W | 21–30.i.2008 (1 ♂, MZUSP).

##### Additional examined material.

**Brazil**, MS [state of Mato Grosso do Sul]: Porto Murtinho (norte), Estrada para Pirizal, km18, 21°33'05"S, 57°45'35"W, 19–31.i.2008, Nihei, S.; • Figueiredo, R. & Almeida, J. (col.) (2 ♀, MZUSP; 2 ♀, NHMW); same data, except: Trilha Fazenda Campo Florido, 21°41'52,0"S, 57°45'57,1"W, Ativa, 11.xii.2013, Lamas & eq. cols. (4 ♀, two dissected, MZUSP).

##### Remarks.

The new species is easily recognized by the characters presented in the key and diagnosis (see above).

##### Distribution.

Brazil (state of Mato Grosso do Sul) (Fig. 19).

##### Etymology.

From the Tupi-guarani *aysú* = love, referring to the male terminalia, which resembles an ideogram of a heart in dorsal view. The species’ name is treated as a noun in apposition.

#### ﻿ Cardiasilus dangeloi sp. nov.

Taxon classificationAnimaliaDipteraAsilidae

10EDCD72-18F8-54D9-A3A0-979C657815B3

https://zoobank.org/1CF4F823-5199-4E5D-9B5F-5145385C99E3

Figs 6 , 7 , 8 , 19

##### Diagnosis.

**Figure 6.**
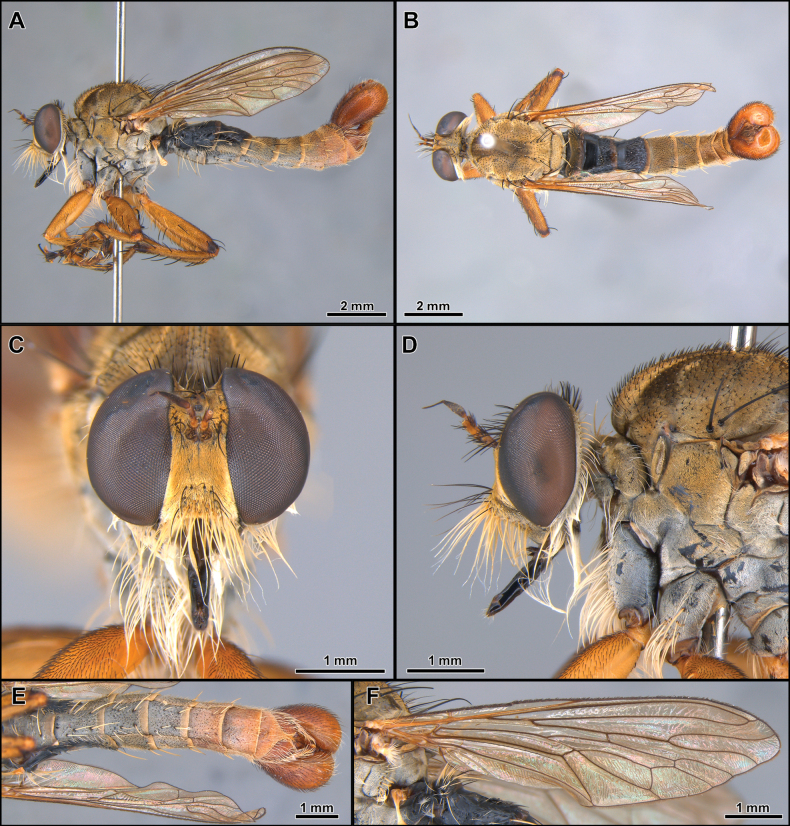
*Cardiasilusdangeloi* sp. nov. (male holotype, before dissection) **A, B** habitus lateral and dorsal, respectively **C** head, anterior view **D** head and thorax, lateral view **E** abdomen, ventral view **F** wing.

**Figure 7.**
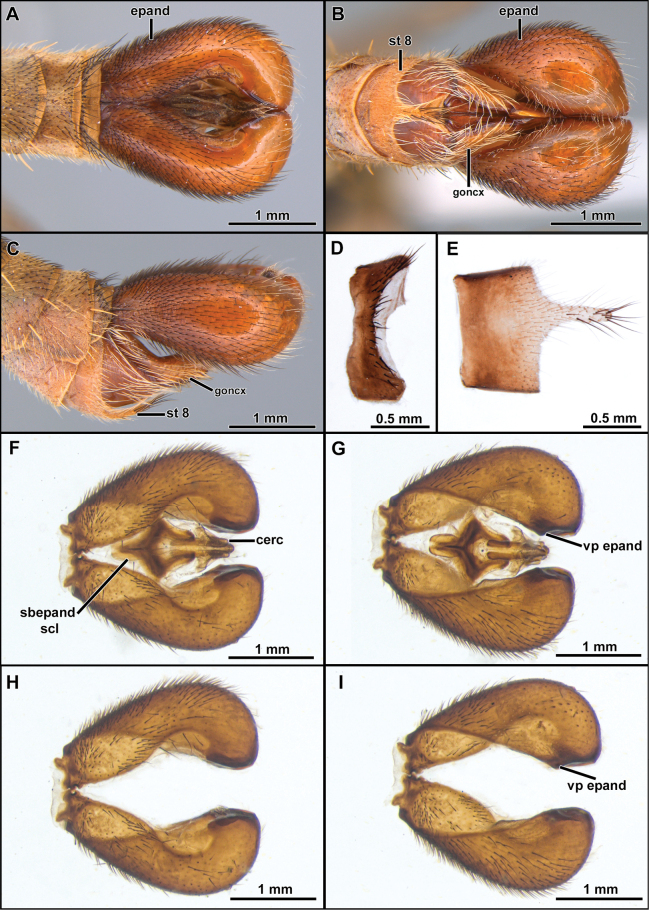
*Cardiasilusdangeloi* sp. nov. (male holotype) **A–C** terminalia in dorsal, ventral, and lateral views, respectively **D** tergite 8 **E** sternite 8 **F, G** dissected terminalia in dorsal and ventral views, respectively **H, I** dissected terminalia without inner appendages in dorsal and ventral views, respectively. Abbreviations: cerc = cercus; epand = epandrium; goncx = gonocoxite; sbepand scl = subepandrial sclerite; st 8 = sternite 8; vp epand = ventral process of epandrium.

**Figure 8.**
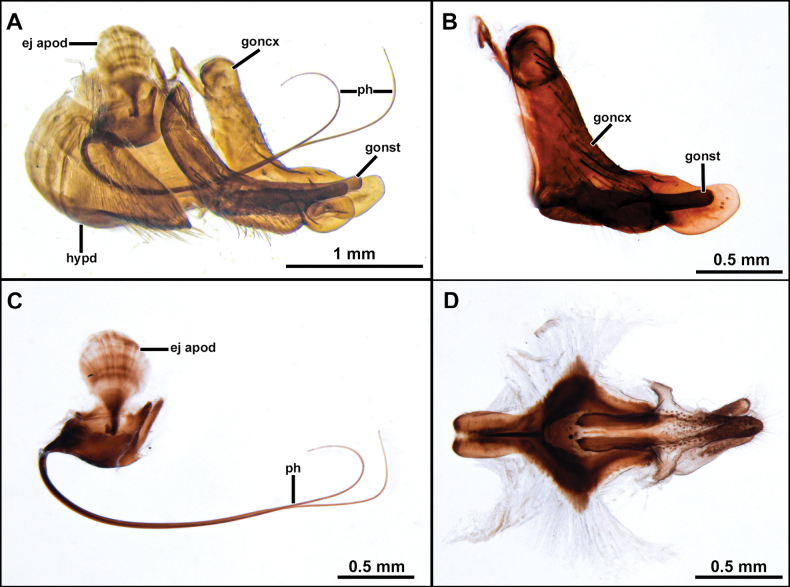
*Cardiasilusdangeloi* sp. nov. (male holotype) **A** inner appendages **B** gonocoxite and gonostylus **C** phallus and ejaculatory apodeme **D** subepandrial sclerite. Abbreviations: ej apod = ejaculatory apodeme; hypd = hypandrium; goncx = gonocoxite; gonst = gonostylus; ph = phallus.

The black postalar macrosetae (Fig. 6B), the epandrium strongly excavated at mid-inner dorsal margin (Fig. 7A, F, H), and the subepandrial sclerite somewhat trapezoidal at middle (Fig. 8D) should promptly distinguish this species from its congeners.

##### Description.

**Male** (Fig. 6A). Body Length: 13.2 mm; wing length: 7.1 mm. ***Head*** (Fig. 6A–D). Similar to *C.aysu* sp. nov. except as noted: ***Thorax*** (Fig. 6A, B, D) postalar macrosetae black. ***Legs*** (Fig. 6B). **Leg I.** Tibia with posterior row of four long black macrosetae from basal 1/4 to apex. **Leg II.** Femur with anterior row of four strong black macrosetae (basalmost white). Tibia with macrosetae wholly black. **Leg III.** Femur with anteroventral row of short, strong, and sparse black macrosetae (basalmost white). ***Terminalia*** (Figs 7, 8). Similar to *C.aysu* sp. nov., except as noted: Epandrium strongly excavated at mid-inner dorsal margin (Fig. 7A, F); subepandrial sclerite somewhat trapezoidal at its middle (Fig. 8D).

**Female.** Unknown.

##### Type examined material.

***Holotype*** • ♂ (MZUSP) labelled: “BRASIL: MS [state of Mato Grosso do Sul]: Porto Murtinho | Faz. São Fernando – km12 | 21°36'30"S, 57°49'02"W | 19–31.I.2008 | Nihei, S., Figueiredo, R. & Almeida, J. | (col.)” “HOLOTYPE | *Cardiasilusdangeloi* | Soares, Camargo & Lamas [red label]”. Holotype condition: Good, left postpedicel broken off, abdomen glued to thorax with remains of glue obscuring pruinosity of tergites one to three, terminalia dissected and stored in microvial at the same pin.

##### Remarks.

The new species is remarkably similar to *C.aysu* sp. nov., differing only in the color of postalar macrosetae black (Fig. 6B), anterior row of macrosetae on mid femur wholly black, only the basalmost macroseta white (Fig. 6A), epandrium strongly excavated at mid-inner dorsal margin (Fig. 7A, F, H) and subepandrial sclerite somewhat trapezoidal at middle (Fig. 8D). In *C.aysu* sp. nov. the postalar macrosetae are yellow (Fig. 2C), the anterior row of macrosetae on the mid femur is white, the mid-inner dorsal margin of the epandrium is weakly excavated (Fig. 3A) and the subepandrial sclerite is somewhat diamond-shaped (Fig. 4D). The epandrium strongly excavated medially at inner margin and the lack of a dorsal preapical process also differs *C.dangeloi* sp. nov. from *C.ruda* sp. nov.

##### Distribution.

Brazil (state of Mato Grosso do Sul) (Fig. 19).

##### Etymology.

The new species is named after Gio D’Angelo (INPA), an artist, photographer, and myrmecologist who is the partner of the first author and deeply passionate about scientific illustration. Gio also kindly created the illustration for Fig. 20.

#### ﻿ Cardiasilus ruda sp. nov.

Taxon classificationAnimaliaDipteraAsilidae

6FED5ABB-577F-536F-A4CB-5F375D3F8B16

https://zoobank.org/00CEB9B1-E2CF-4C9B-B998-C8305DA907BB

**Figure 9.**
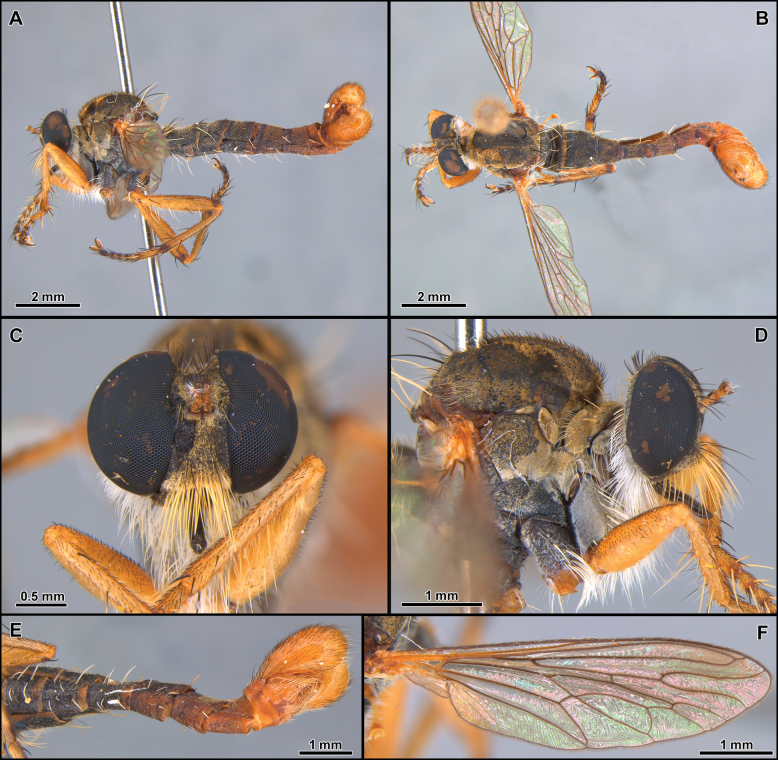
*Cardiasilusruda* sp. nov. (male holotype, before dissection) **A, B** habitus lateral and dorsal, respectively **C** head, anterior view **D** head and thorax, lateral view **E** abdomen, ventral view **F** wing.

**Figure 10.**
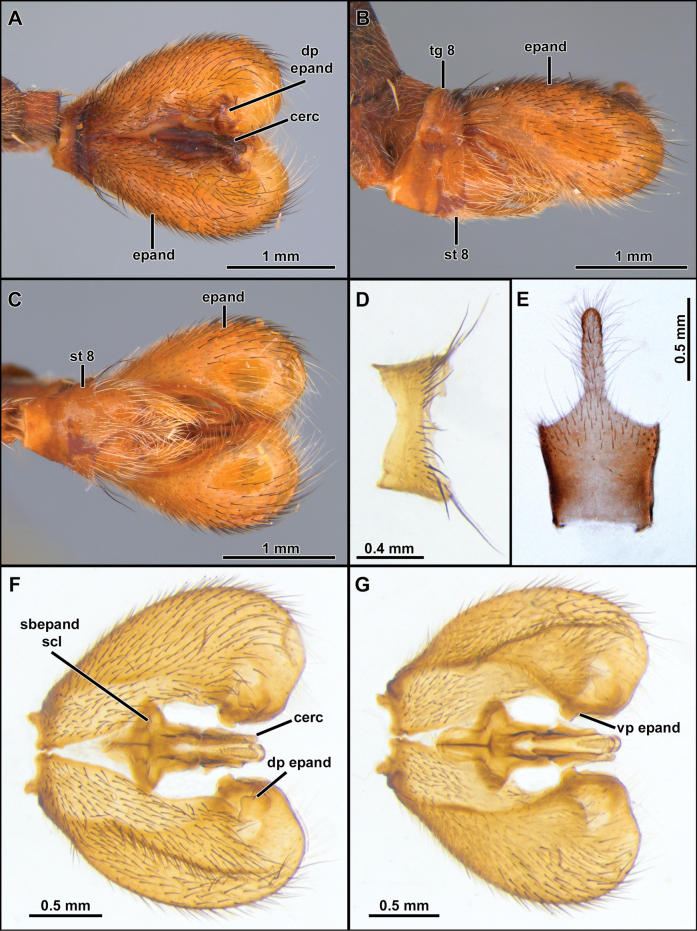
*Cardiasilusruda* sp. nov. (male holotype) **A–C** terminalia in dorsal, lateral, and ventral views, respectively **D** tergite 8, **E** sternite 8 **F, G** dissected terminalia in dorsal and ventral views, respectively. Abbreviations: cerc = cercus; epand = epandrium; dp epand = dorsal process of epandrium; goncx = gonocoxite; sbepand scl = subepandrial sclerite; st 8 = sternite 8; tg 8 = tergite 8; vp epand = ventral process of epandrium.

**Figure 11.**
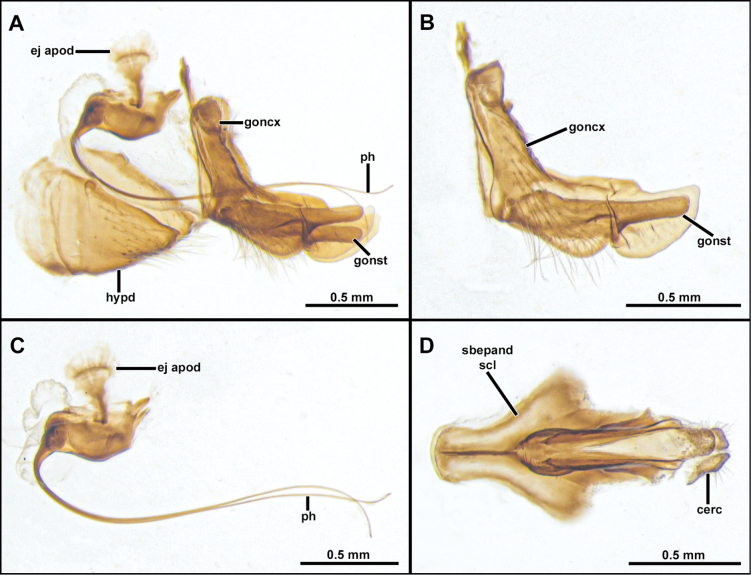
*Cardiasilusruda* sp. nov. (male holotype) **A** inner appendages **B** gonocoxite and gonostylus **C** phallus and ejaculatory apodeme **D** subepandrial sclerite. Abbreviations: cerc = cercus; ej apod = ejaculatory apodeme; hypd = hypandrium; goncx = gonocoxite; gonst = gonostylus; ph = phallus.

Figs 9 , 10 , 11 , 19

##### Diagnosis.

The inner margin of epandrium with a short, dorsal, sub-triangular, pre-apical process followed by a short, ventral, sub-rectangular, anteriorly curved, apical process (Fig. 10A, F, G) separates this species from its congeners.

##### Description.

**Male** (Fig. 9A). Body Length: 10.3 mm; wing length: 5.7 mm. ***Head*** (Fig. 9A–D). Similar to *C.aysu* sp. nov. except as noted: ***Thorax*** (Fig. 9A, B, D). Two pairs of dorsocentral macrosetae. ***Terminalia*** (Figs 10, 11). Epandrium with a short, dorsal, sub-triangular, pre-apical process followed by a short, ventral, sub-rectangular, anteriorly curved, apical process (Fig. 10A, F, G). Subepandrial sclerite goblet-shaped (Fig. 11D).

**Female.** Unknown.

##### Type examined material.

***Holotype*** • ♂ (MZUSP) labelled: “PARAGUAI | Colônia Piraretá [ca 25°30'09.2"S, 56°59'00.1"W] | 25.xii.1971” “HOLOTYPE | *Cardiasilusruda* | Soares, Camargo & Lamas [red label]”. Holotype condition: Both postpedicel and mid legs broken off, terminalia dissected and stored in microvial at the same pin.

##### Remarks.

The new species is remarkably similar to *C.aysu* sp. nov., differing only in the shape of epandrium with a short, dorsal, sub-triangular, pre-apical process followed by a short, ventral, sub-rectangular, anteriorly curved, apical process (Fig. 10A, F, G). The epandrium lacks such processes at dorsal inner margin in *C.aysu* sp. nov., and *C.dangeloi* sp. nov.

##### Distribution.

Paraguay (department of Cordillera) (Fig. 19).

##### Etymology.

Rudá is the deity of love in the pantheon of deities of Tupi-Guarani culture. It alludes to the male epandria that resembles an ideogram of a heart (Fig. 10A). Treated as a noun in apposition.

#### ﻿ Martintella

Taxon classificationAnimaliaDipteraAsilidae

Artigas

1E857403-AE4C-512D-8103-6CC604C7E609

Fig. 12

Martintella Artigas, 1996: 75 (*nomen novum* for Martinella Artigas & Papavero, 1995). Type species: Asiluslestes Williston, 1901 (original designation). Type locality: Mexico, Guerreiro, Chilpancingo

##### Remarks.

**Figure 12.**
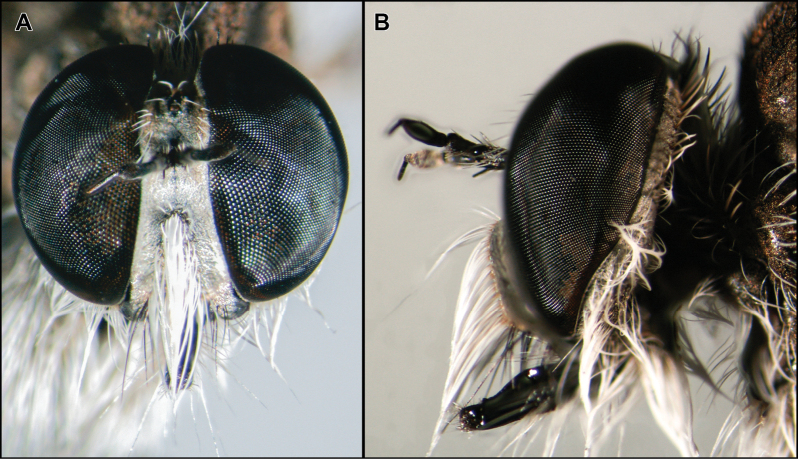
*Martintella* sp. (male from Mexico) **A, B** Head in anterior and lateral views, respectively. Photographs provided by Frida Bello (INECOL).

The genus *Martintella* was erected to accommodate the species *Asiluslestes* Williston, 1901, at the time placed in *Wilcoxius*. Subsequently, Scarbrough (2010) described the second species, *Martintellaelliptica* Scarbrough, 2010, from Trinidad and Tobago. Finally, Vieira et al. (2014) described two new species from Costa Rica, and commented on the possibly dubious position of *Martintellaelliptica* in the genus. According to Fisher (2009), species of *Martintella* are similar to those of *Wilcoxius*. However, *Martintella* is easily recognized by the frons with convergent slopes (Fig. 12A), mystax restricted to middle of face, resembling a mohawk (Fig. 12A, B), sternites lacking macrosetae, and phallus completely concealed versus frons with parallel slopes (Fig. 18D), mystax not restricted to the middle of face and not resembling a mohawk (Fig. 18D), sternites two to five with one to two pairs of macrosetae (Fig. 18E), and phallus exposed (Fig. 18F).

##### Distribution.

Costa Rica, Mexico, and Trinidad and Tobago.

#### ﻿ Myaptex

Taxon classificationAnimaliaDipteraAsilidae

Hull

8B2F2EFA-88E3-5167-A023-AC47CCEF9DC4

Figs 13 , 14

Myaptex Hull, 1962 (2): 508. Type species, Myaptexhermanni Hull, 1962 (original designation). Type locality: Chile, Concepción.

##### Remarks.

**Figure 13.**
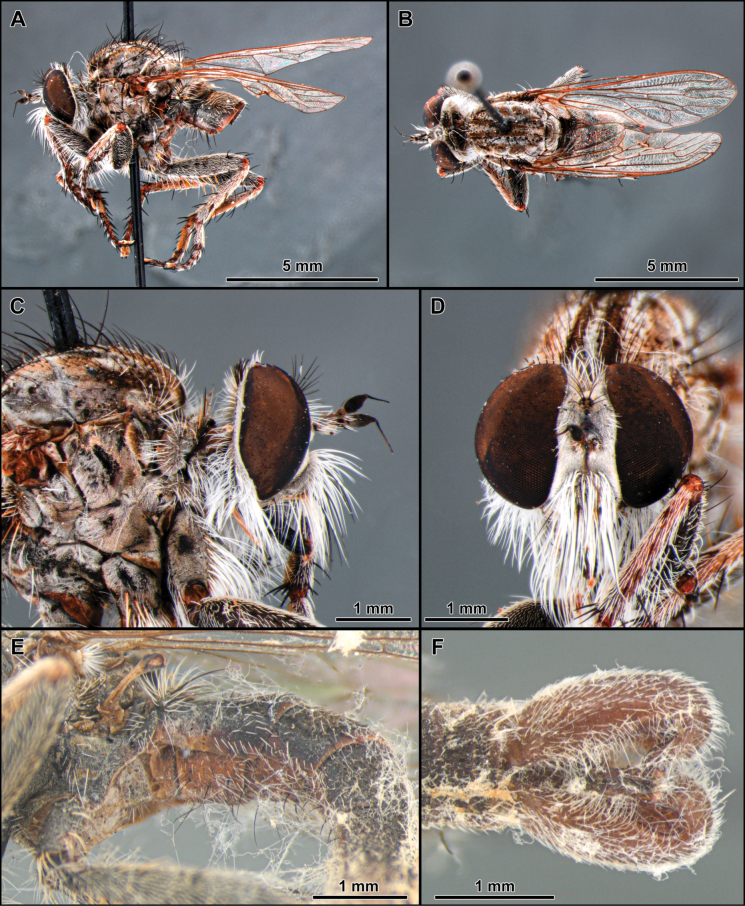
*Myaptexbrachyptera* (Philippi, 1865) (identified male from Chile) **A, B** habitus lateral and dorsal, respectively, **C** head and thorax, lateral view **D** head, anterior view **E** abdomen, lateroventral view **F** terminalia, dorsal view.

**Figure 14.**
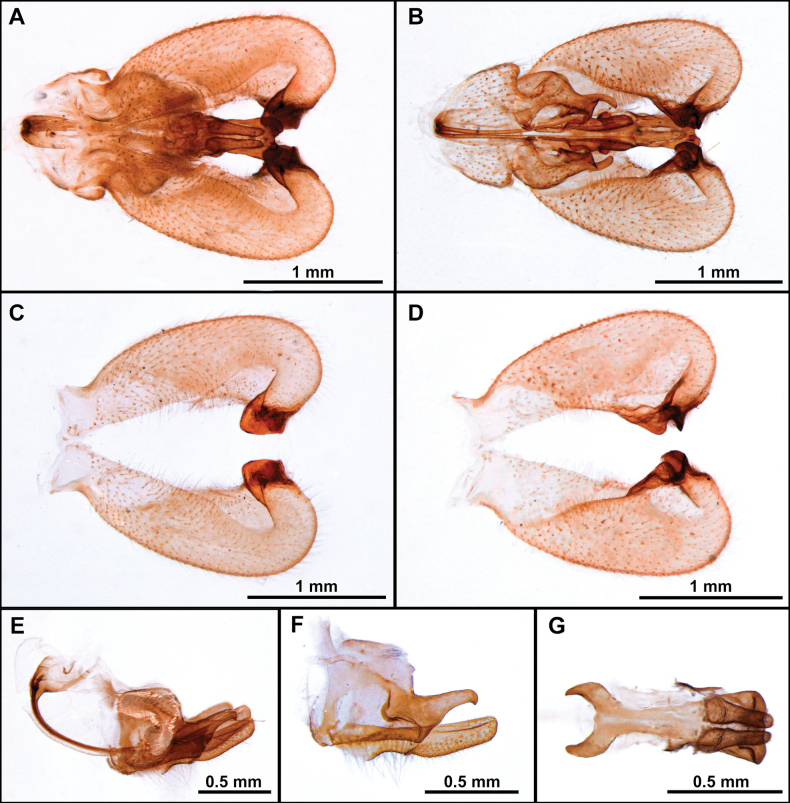
*Myaptexbrachyptera* (Philippi, 1865) (identified male from Chile) **A, B** Terminalia in dorsal and ventral views, respectively **C, D** terminalia without inner appendages in dorsal and ventral views, respectively **E** inner appendages, lateral view **F** gonocoxite and gonostylus, lateral view **G** cercus and subepandrial sclerite, dorsal view.

**Figure 15.**
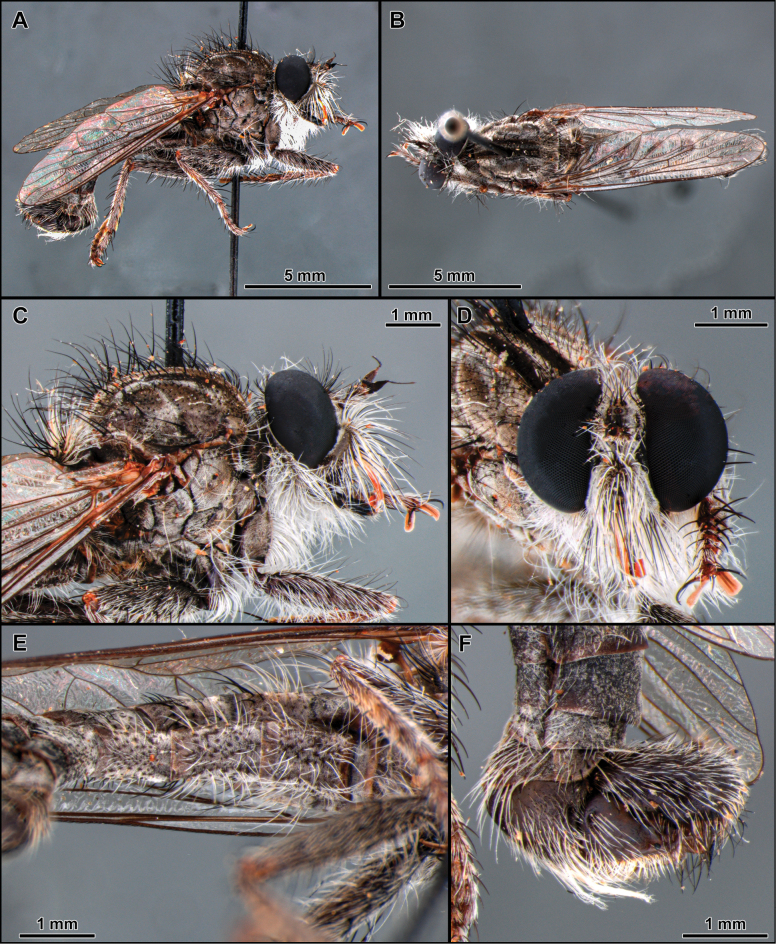
*Myaptexariavexillaria* Artigas, 1970 (male paratype) **A, B** habitus lateral and dorsal, respectively **C** head and thorax, lateral view **D** head, anterior view **E** abdomen, ventral view **F** terminalia, lateral view.

*Myaptex* is endemic to Chile and comprises only two species (Papavero 2009). The genus resembles *Myaptexaria* by the face distinctly gibbous, scutum with conspicuous rows of acrostichal setae and anterior dorsocentral macrosetae present, but can be easily segregated by the scutellar disc only with scattered, long setae (Fig. 13B); normally two to four black apical scutellar macrosetae; male terminalia with epandria strongly inflated, their apices curved in apically (Figs 13F, 14A–D) in *Myaptex* versus scutellar disc with two tufts of abundant, proclinate, long setae (Fig. 15B, C), from two to several apical scutellar macrosetae (sometimes mixed black and white) (Fig. 15C), male terminalia with epandria not inflated, their apices blunt and not curved at apex (Fig. 15F) in *Myaptexaria*. *Myaptex* is also superficially similar to *Cardiasilus* gen. nov. (see remarks under *Cardiasilus* gen. nov.), but the only feature they both share is the inflated male epandria (a feature that probably arose independently *de novo* in both genera).

##### Distribution.

Chile.

##### Examined material.

Chile, Maule, Cauquenes, 25.i.1955, L.E. PENA (1 ♂, dissected MZUSP).

#### ﻿ Myaptexaria

Taxon classificationAnimaliaDipteraAsilidae

Artigas & Papavero

5988F332-D0EC-55EC-AA22-E8BD545DCAA1

Fig. 15

Myaptexaria Artigas & Papavero, 1995: 58. Type species: Myaptexvexillaria Artigas, 1970 (original designation). Type locality: Chile, Coquimbo, Vicuña.

##### Remarks.

*Myaptexaria* was erect to accommodate three species placed in *Myaptex*: *M.acuta* (Artigas), *M.vexillaria* (Artigas) (type-species) and *M.virilis* (Artigas). Both genera can be easily segregated by the shape of male epandria (as discussed above under remarks of *Myaptex*).

##### Distribution.

Chile.

##### Examined material.

Chile, Coquim. [Coquimbo] Had [Hacienda] Illapel, 600–1000 m, 24–25.x.1954, L.E. PENA (1 ♂, 2 ♀, MZUSP).

#### ﻿ Papaverellus

Taxon classificationAnimaliaDipteraAsilidae

Artigas & Vieira

AD9D28DB-1D4F-53C2-A892-B5168DFE56F0

Papaverellus Artigas & Vieira, 2014: 283. Type species: Papaverellusaureocingulatus Artigas & Vieira, 2014 (original designation). Type locality: Brazil, state of Pará, Belém.

##### Remarks.

The monotypic genus *Papaverellus* shares one similarity with *Cardiasilus* gen. nov., the bifurcation of veins *R_4_* and *R_5_* before the apex of discal cell (Artigas and Vieira 2014: fig. 5). However, both genera can be distinguished by the abdominal sternites without macrosetae (Artigas and Vieira 2014: fig. 1) and epandria not inflated and flat ventrally, resembling a goat hoof (Artigas and Vieira 2014: fig. 1) in *Papaverellus*, whereas *Cardiasilus* gen. nov. has the abdominal sternites with at least three pairs of macrosetae (Figs 2H, 6E, 9E) and male epandria inflated resembling the ideogram of a heart (i.e., Figs 2B, H, 3A, C).

##### Distribution.

Brazil (states of Pará and Piauí).

#### ﻿ Rhadinosoma

Taxon classificationAnimaliaDipteraAsilidae

Artigas

15F0D43E-D5B6-57A1-8357-3C95E64646FA

Fig. 16

Rhadinosoma Artigas, 1970: 346. Type species: Rhadinosomacalderense Artigas, 1970 (original designation). Type locality: Chile, Atacama.

##### Remarks.

**Figure 16.**
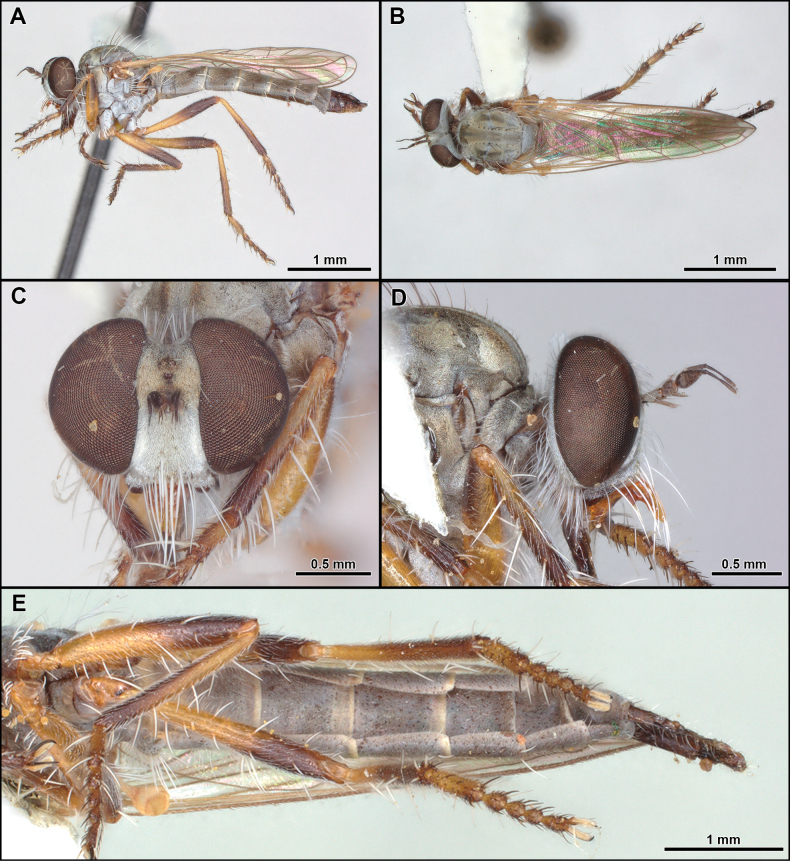
*Rhadinosoma* sp. (female from Chile) **A, B** habitus lateral and dorsal, respectively **C** head, anterior view **D** head and thorax, lateral view **E** abdomen, ventral view. Photographs provided by Denise Montelongo (CAS).

The monotypic genus *Rhadinosoma* is also known to occur only in Chile. It can be easily segregated from the other genera of the *Myaptex* group by the characteristics presented in the key above, mainly the mystax composed of few sparse macrosetae (Fig. 16C, D) and the vein *R_4_* ending at wing apex (Fig. 16B).

##### Distribution.

Chile.

##### Examined material

(based on photographs). Chile, Atacama, Prov. 70 km. S. Copiapo, X–5–1966, E.I. Schlinger, M.E. Irwin, dune Assoc. (1 ♀, CAS).

#### ﻿ Scarbroughia

Taxon classificationAnimaliaDipteraAsilidae

Papavero

5F37ECD2-4EE9-5B0B-9A39-95A1E8AD806F

Fig. 17

Scarbroughia Papavero, 2009: 46 (nomen novum for Furcilla Martin, 1975). Type species: Furcilladorothyae Martin, 1975 (original designation). Type locality: Mexico, Sonora.

##### Remarks.

**Figure 17.**
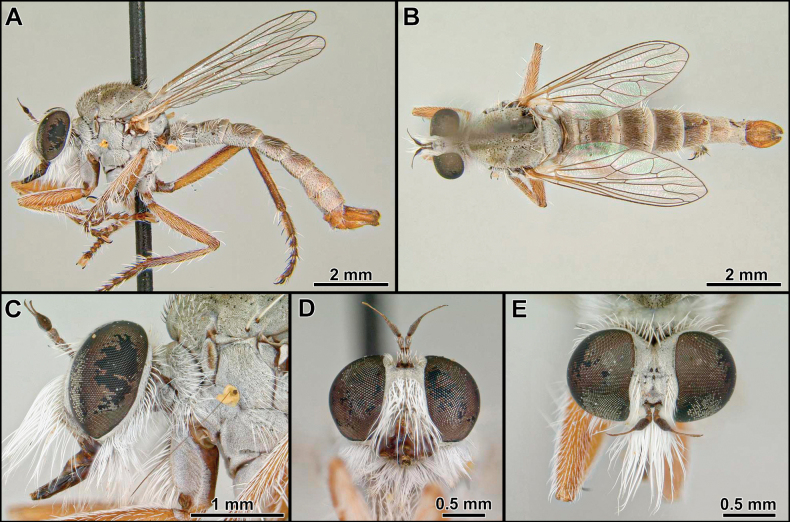
*Scarbroughiadorothyae* (Martin, 1975) (male holotype) **A, B** habitus lateral and dorsal, respectively **C** head, lateral view **D** face, anterior view **E** frons, anterior view. Photographs provided by Christopher Grinter (CAS).

The genus *Scarbroughia* comprises two species from Sonora, Mexico, and can be segregated by the mystax composed of abundant and dense macrosetae (Fig. 17C, D), face flat (Fig. 17C), well-developed anterior dorsocentral macrosetae absent (Fig. 17A) and the vein *R_4_* ending above wing apex (Fig. 17B).

##### Distribution.

Mexico.

##### Examined material

(based on photographs). Mexico, 11 mi. South Navojos, Sonora, Hy. 15 Km. 1766, Sept. 3, 1962, Holotype Furcilladorothyae Cash. H. Martin, California Academy of Sciences, Type No. 12579 (1 ♂, CAS).

#### ﻿ Wilcoxius

Taxon classificationAnimaliaDipteraAsilidae

Martin

64E40018-8B2A-5A89-BBE8-41C1F18DFBFE

Fig. 18

Wilcoxius Martin, 1975: 71. Type species: Wilcoxiustruncus Martin, 1975 (original designation). Type locality: Mexico, Veracruz.

##### Remarks.

**Figure 18.**
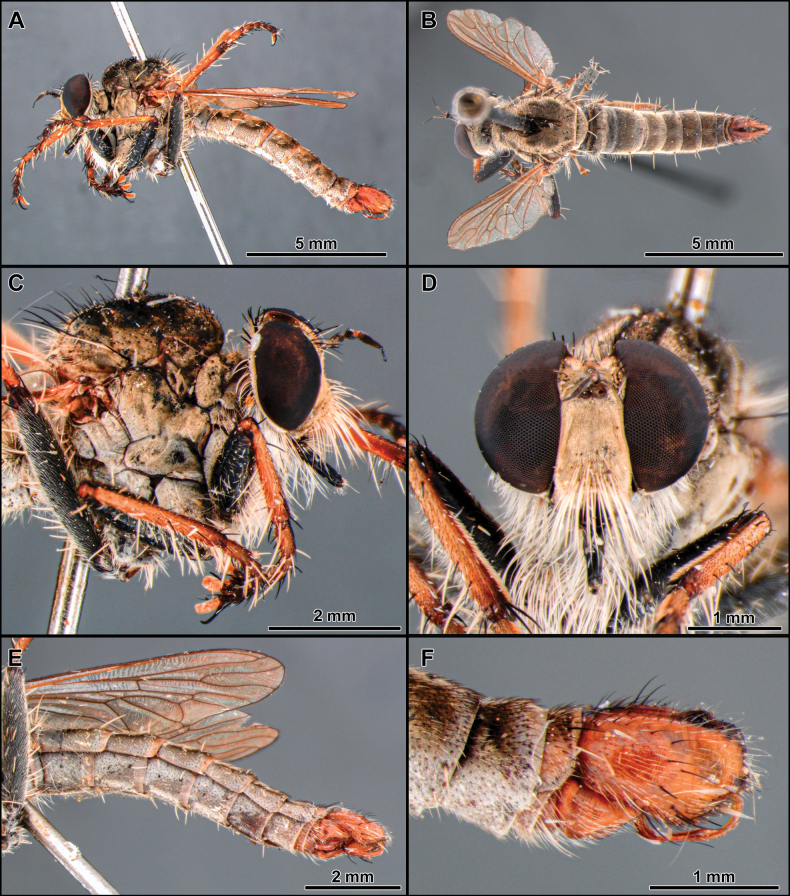
*Wilcoxiustruncus* (Martin, 1975) (male holotype) **A, B** habitus lateral and dorsal, respectively **C** head and thorax, lateral view **D** head, anterior view **E** abdomen, lateroventral view **F** terminalia, lateral view.

According to Fisher (2009) the genus *Wilcoxius* is similar to *Martintella* (see remarks under *Martintella*), but both genera can be promptly distinguished by frons with parallel slopes (Fig. 18D), mystax restricted to the lower 1/2 of face and composed of sparse setae (Fig. 18C, D), abdominal sternites with macrosetae (Fig. 18E) and phallus exposed (Fig. 18F) versus frons with convergent slopes (Fig. 12A), mystax occupying 2/3 of face and restricted to the middle of face, resembling a mohawk (Fig. 12A, B), abdominal sternites without macrosetae (Vieira et al. 2014: figs 1, 17, 29) and phallus concealed (Vieira et al. 2014: figs 1, 17, 29) in *Martintella*.

##### Distribution.

Cuba, Dominican Republic, El Salvador, Guatemala, Honduras, Mexico, and Nicaragua.

##### Examined material.

Mexico, 40 mi E, Tehuantepec, Oax. 3.viii.1967, Altitude 500ft., RH & EM Painter collectors, PARATYPE, Wilcoxiustruncus, Chas. H. Martin (1 ♂, MZUSP).

**Figure 19.**
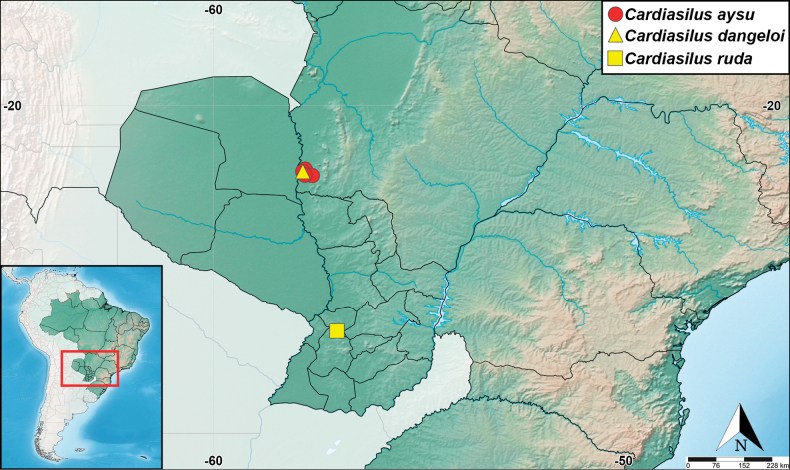
Known geographical distribution of *Cardiasilus* gen. nov. species.

**Figure 20.**
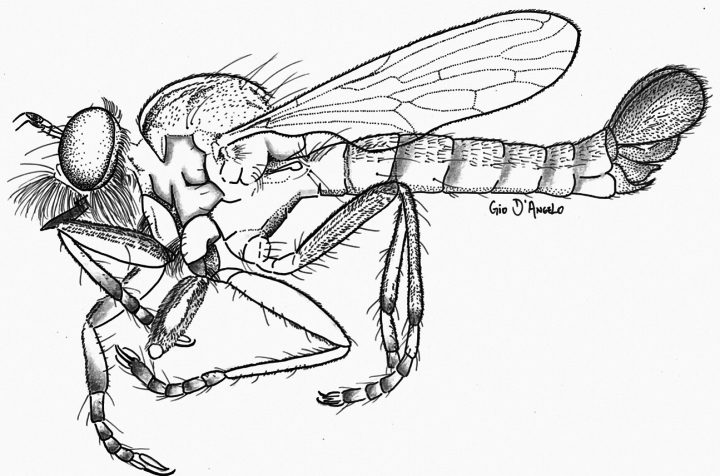
Illustration of *Cardiasilusdangeloi* gen. nov. et sp. nov.

## ﻿Discussion

Using the adapted key for genera of the *Myaptex* group provided in Camargo et al. (2022)*Cardiasilus* gen. nov. will key out to *Papaverellus* due to the bifurcation of the veins *R_4_* and *R_5_* before the apex of the discal cell (on couplet 5 of Camargo et al. (2022) key, where it says “Mystax with abundant bristles occupying 3/4 of face” it should be “Mystax occupying the entire facial gibbosity”). Despite of this similarity on the wing pattern, specimens herein studied can be easily segregated from *Papaverellus* due to the inflated heart ideogram-shaped epandria (flat ventrally, and resembling a goat hoof in *Papaverellus*) and the presence of paired well-developed macrosetae on the sternites 2–7 (sternites without well-developed macrosetae in *Papaverellus*).

Regarding the short recurrent (stump) vein on *R_4_* that arises close to the origin of *R_5_* and is presented here as a diagnostic character for *C.ruda* sp. nov., caution is warranted in interpreting this feature. Additional specimens should be collected and examined to verify the diagnostic significance of this character, as the presence or absence of stump veins often demonstrates high variation within the Asilinae genera or species. To illustrate this scenario in the *Myaptex* group, the male paratype of *Martintellaelliptica*, provides an example where the recurrent vein is present on the right wing but absent on the left wing (see Vieira et al. 2014: 453, fig. 8). Similarly, in *Apulvillasilus*, the recurrent vein is absent on the male holotype but present on the female paratype (see Camargo et al. 2022: 4, 7, figs 2, 6, 19).

*Cardiasilus* gen. nov. is until now known only from the localities of Porto Murtinho, Mato Grosso do Sul state, Brazil, and Colonia Piraretã, Cordillera Department, Paraguay. Both localities are in the eastern side of the Paraguay river in a region that has been historically denominated as part of the “eastern” or “humid chaco”. This terminology is currently being considered an unsuitable designation that does not reflect the reality of this region, shaped of a mosaic of ecoregions with a number of widely different florist communities dominated by semi-dry and semi-deciduous seasonal forests with interspersed intrusions and islands of “chaquenian”, vegetation elements delimited mainly by the different types of soil (Prado et al. 1992; Prado 1993; Avila et al. 2018; Garcete-Barrett *in litt.*).

The Piraretã region is located in the “Cordillera de Los Altos” ecoregion (Avila et al. 2018) and is currently highly anthropized. However, its original vegetation must have been composed mainly of elements from the semi-deciduous Paraná forest, with rocky outcrops in the Saltos de Piraretã region and with “chaquenian” influence in the lower parts (Garcete-Barrett *in litt.*). The portion of the “humid chaco” assigned to Brazil is restricted to the surroundings of Porto Murtinho, which is also a place where different floristic stocks meet (Prado et al. 1992; Prado 1993). In the present study, specimens were collected in areas with open Arborized Stepic Savannah. It is herein postulated that the new genus might be endemic of the mosaic “humid chaco” region along the Paraguay river. Additionally, several Malaise samples collected in localities in Goiás state (which borders) Mato Grosso do Sul state containing huge amounts of Asilidae specimens were analyzed by the first author and not even a single specimen of the new genus was found reinforcing the endemism assumption.

The present paper represents an important contribution to the knowledge of the Chaco biome biodiversity. Recent taxonomic studies of the Diptera fauna in the Chaco domain (Lamas et al. 2015; Riccardi et al. 2018; Quevedo et al. 2024) in Porto Murtinho (Mato Grosso do Sul, Brazil) have revealed a high degree of endemism among Diptera in this region. Many of the samples collected (Lamas et al. 2023) represent new taxa that are not merely new species within known genera; their striking divergence in certain morphological traits suggests the establishment of new genera. This study includes the first recorded instance for the family Asilidae, with all examined material sourced from the humid chaco regions of Brazil and Paraguay. The identification of these previously unknown and unusual Diptera emphasizes the urgent need for international efforts to study and preserve the South American Chaco.

## Supplementary Material

XML Treatment for Apulvillasilus

XML Treatment for Atractocoma

XML Treatment for Cardiasilus

XML Treatment for Cardiasilus aysu

XML Treatment for Cardiasilus dangeloi

XML Treatment for Cardiasilus ruda

XML Treatment for Martintella

XML Treatment for Myaptex

XML Treatment for Myaptexaria

XML Treatment for Papaverellus

XML Treatment for Rhadinosoma

XML Treatment for Scarbroughia

XML Treatment for Wilcoxius

## ﻿Additional information

### Author ORCIDs

Matheus M. M. Soares https://orcid.org/0000-0002-2355-1441

Alexssandro Camargo https://orcid.org/0000-0001-7408-7223

Carlos J. E. Lamas https://orcid.org/0000-0002-7750-590X

### Data availability

All of the data that support the findings of this study are available in the main text.
